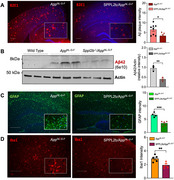# Inhibiting of Presenilin‐Like Peptidase SPPL2b Reduces Neuroinflammation, Amyloid Pathology, and Synaptic Loss in Alzheimer's Disease: A Promising Therapeutic Target

**DOI:** 10.1002/alz70855_102660

**Published:** 2025-12-23

**Authors:** Jack Badman, Bjorn Bakker, Rajnish Kumar, Eileen Mac Sweeney, Beatriz Pacheco Sánchez, Wenjun Li, Per Nilsson, Simone Tambaro

**Affiliations:** ^1^ Karolinska Institutet, Solna, Sweden; ^2^ Indian Institute of Technology, Varanasi, India

## Abstract

**Background:**

Signal peptidyl peptidase 2b (SPPL2b) is an intramembrane peptidase, from the same family of presenilins, and is predominantly expressed in the brain. SPPL2b is involved in the cleavage of various transmembrane proteins linked to the pathophysiology of Alzheimer's disease (AD), such as BRI2, TNFα, Cleac7a, and TMEM106B. Recently, we demonstrated that SPPL2b inhibition reduces the processing of amyloid precursor protein (APP), resulting in decreased production of amyloid‐beta 42 (Aβ42) and amyloid‐beta 40 (Aβ40). In this study, we aimed to explore the therapeutic potential of SPPL2b by deleting the SPPL2b gene in the *App^NL‐G‐F^* knock‐in AD mouse model, and we aimed, since no specific inhibitors of SPPL2b currently exist, to identified potential SPPL2b inhibitory compounds through *in silico* screening.

**Method:**

To investigate the therapeutic potential of SPPL2b inhibition, we developed a novel AD mouse model by crossbreeding *App^NL‐G‐F^* knock‐in mice with SPPL2b‐deficient mice. Brain samples from these mice were analyzed using western blotting, immunofluorescence, and Golgi staining. To identify potential inhibitors of SPPL2b we screened the Vitas‐M commercial library.

**Result:**

We observed a significant reduction in Aβ plaque deposition in the brains of SPPL2b knockout (KO) / *App^NL‐G‐F^* mice at both 4 and 12 months of age. Additionally, cortical and hippocampal gliosis were markedly reduced in these mice (Figure 1). At 12 months, Golgi staining analysis showed that SPPL2b deletion protected against synaptic loss in *App^NL‐G‐F^* mice, as evidenced also by the rescue of the postsynaptic density marker PSD95 in SPPL2b KO / *App^NL‐G‐F^* mice. Moreover, a decrease in phospho‐tau intensity was observed. Importantly, we have identified 100 potential SPPL2b inhibitory compounds.

**Conclusion:**

Our findings underline the critical role of SPPL2b in the development of Aβ pathology in AD. In a global context where innovative strategies to prevent and mitigate AD progression are urgently needed, this study highlights SPPL2b as a promising therapeutic target. Futhermore, the identified compounds are now undergoing *in vitro* evaluation and the most promising inhibitors will be tested *in vivo* to assess their effects on AD pathology.